# Epileptogenic Zone Location of Temporal Lobe Epilepsy by Cross-Frequency Coupling Analysis

**DOI:** 10.3389/fneur.2021.764821

**Published:** 2021-11-16

**Authors:** Xiaotong Liu, Fang Han, Rui Fu, Qingyun Wang, Guoming Luan

**Affiliations:** ^1^Department of Dynamics and Control, Beihang University, Beijing, China; ^2^College of Information Science and Technology, Donghua University, Shanghai, China; ^3^Beijing Key Laboratory of Epilepsy, Sanbo Brain Hospital, Capital Medical University, Beijing, China

**Keywords:** cross-frequency coupling, temporal lobe epilepsy, SEEG, functional network, epileptogenic zone

## Abstract

Epilepsy is a chronic brain disease with dysfunctional brain networks, and electroencephalography (EEG) is an important tool for epileptogenic zone (EZ) identification, with rich information about frequencies. Different frequency oscillations have different contributions to brain function, and cross-frequency coupling (CFC) has been found to exist within brain regions. Cross-channel and inter-channel analysis should be both focused because they help to analyze how epilepsy networks change and also localize the EZ. In this paper, we analyzed long-term stereo-electroencephalography (SEEG) data from 17 patients with temporal lobe epilepsy. Single-channel and cross-channel CFC features were combined to establish functional brain networks, and the network characteristics under different periods and the localization of EZ were analyzed. It was observed that theta–gamma phase amplitude coupling (PAC) within the electrodes in the seizure region increased during the ictal (*p* < 0.05). Theta–gamma and delta–gamma PAC of cross-channel were enhanced in the early and mid-late ictal, respectively. It was also found that there was a strong cross-frequency coupling state between channels of EZ in the functional network during the ictal, along with a more regular network than interictal. The accuracy rate of EZ localization was 82.4%. Overall, the combination of single-channel and multi-channel cross-band coupling analysis can help identify seizures and localize EZ for temporal lobe epilepsy. Rhythmic coupling reveals a relationship between the functional network and the seizure status of epilepsy.

## Introduction

Epilepsy is one of the most common neurological disorders, with ~70 million patients worldwide. It is a chronic brain dysfunction syndrome caused by multiple etiologies and is characterized by seizures, transient, and sudden brain dysfunction caused by repeated super-synchronous discharges in clusters of nerve cells in the brain (1). Although pharmacotherapy is an effective treatment option for most patients, nearly one-third of the patients with epilepsy develop resistance to medication, which we refer to as refractory epilepsy (2). The common treatment for this group of patients is surgical excision of the epileptogenic zone. However, some patients continue to have seizures after surgery, mainly because the epileptogenic zone was not completely removed, thus leading to secondary seizures. The underlying seizure mechanism is worthy of investigation to effectively control and treat epilepsy.

Electroencephalography (EEG) is one of the important tools to identify epileptic foci and capture the changes in electrical signal during seizures. Common types of EEG are scalp electroencephalography (scalp EEG), electrocorticography (ECoG), and intracranial electroencephalography (iEEG) with high temporal resolution but low spatial resolution (3). In 1959, Bancaud applied the stereotactic technique to epilepsy surgery, named stereo-electroencephalography (SEEG), to study the spatio-temporal dynamics of epileptic discharges based on clinical features and with high anatomical precision (4). SEEG can record electrical displacements in cortical and subcortical brain regions, and is better at recording the spread of seizures than traditional electroencephalography. Moreover, rich frequency band information in the signal has received much attention, and different rhythmic oscillations have different contributions to brain function, depending on the specific time and location where the rhythm appears. Low-frequency EEG oscillations may represent the concerted activity of large-scale neuronal networks in the brain, whereas high-frequency oscillations may mainly reflect the activity of local neuronal populations (5).

The most direct way to observe changes during seizure is to extract the frequency or time-frequency analysis of the signal to classify seizure states and regions (6–9). However, the frequency is not necessarily independent; it was found that the oscillations of neural network with different frequencies can influence each other. Cross-frequency coupling (CFC), which is a dynamic interaction between neural oscillations in different frequency bands, is closely related to memory, perception, and other brain functions. It provides a new perspective on a variety of physiological characteristics (10, 11). The common types of CFC in EEG data analysis are phase–amplitude coupling (PAC), phase–phase coupling (PPC) (12), and amplitude–amplitude coupling (AAC) (13). In the rat hippocampus, PAC can be observed in the region between theta and gamma, and the feature is also present in the human hippocampus and cortex (14). It has been shown that PAC can be used for studies of working memory and also for labeling seizures (15, 16). As research on CFC has evolved, it has been used to identify brain states and task states in diseases such as epilepsy, Alzheimer's disease (17), and Parkinson's disease (18).

In epilepsy, the application of CFC is primarily focused on the discussion of coupling relationships between different frequency band pairs for phase, amplitude, and other information, to help classify seizure state, predict postoperative outcomes, and coupling characteristics of seizure regions (19–21). The point focuses on changes in CFC within channels. However, the brain is a complex system that works in concert, and the transmission of information requires coordination between neuronal populations. The epileptogenic region is not confined to a single area but may comprise a group of directly interrelated areas (22). Single-channel coupling analysis cannot provide these details. Multi-channel CFC can be correlated to understand how information is transferred between different brain regions. Such studies are currently focused on memory cognition, and it has been observed in networks constructed using CFC that storage in working memory increases the efficiency of network connectivity and information transfer (16). Moreover, the size of the cross-regional PAC (xPAC) predicted success in memory encoding, which may be a mechanism that represents a coordinated relationship between brain regions (23). However, few studies have examined the extraction of network features from cross-channel coupling to observe changes in coupling relationships between regional channels during seizures. We prefer to effectively identify EZ channels and seizure propagation processes from all the detected channels in patients than to discuss coupling feature changes within local brain regions. A problem worth investigating is how to use single-channel and cross-channel CFC to construct functional brain networks to help locate EZ.

In this study, cross-frequency PAC analysis of long-term brain activity was performed in 17 patients with temporal lobe epilepsy. The PAC characteristics within all electrode channels were analyzed without marking the specific seizure times and medial or lateral brain regions. Cross-channel PAC was then calculated based on single-channel analysis. The brain functional network connectivity matrix was derived from the cross-channel cross-band coupling matrix to examine the network characteristics under different periods. The location of the epileptogenic zone and the changing pattern of information transmission in the network during the seizure were analyzed by complex graph theory to aid diagnosis and treatment.

## Materials and Methods

**Figure 1:**
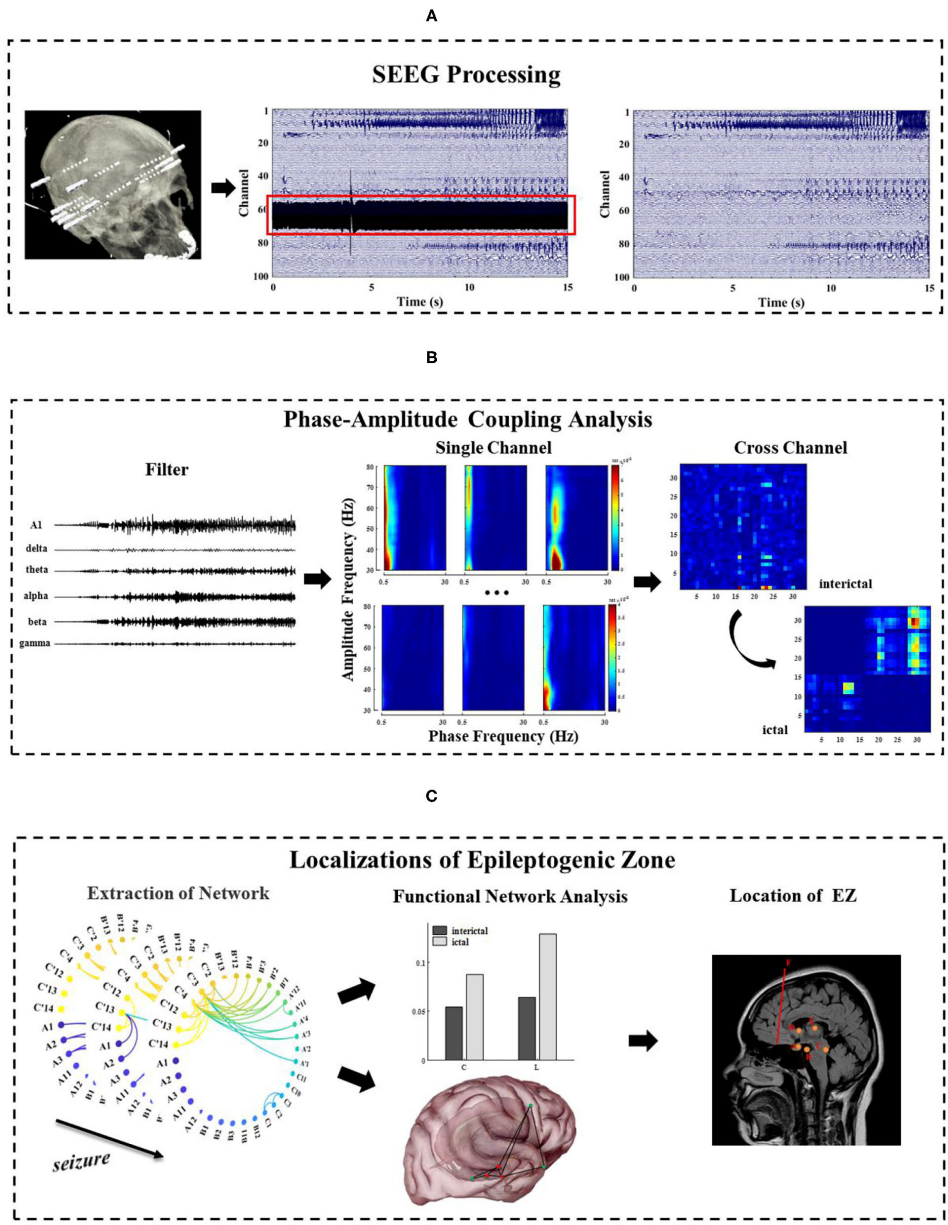
The general framework of methods in this study. **(A)** Pre-process of long-term SEEG data, including denoising and removal of bad channels; **(B)** Extraction of the phase and amplitude information of the different frequency bands (0.5~80 Hz) and calculation of the single-channel and multi-channel phase–amplitude coupling (PAC) under different seizure periods; **(C)** Analysis of the network characteristics and EZ localization by extracting functional networks based on cross-band coupling analysis. This figure is only a schematic diagram. The electrode A–F labeled areas vary for each patient due to patient specificity.

This section focuses on the methods, which are categorized into three major parts. First, the SEEG recording is preprocessed (Figure 1A). Second, frequency information (0.5~80 Hz) is extracted, and single-channel and multi-channel PAC characteristics are calculated during the seizure (Figure 1B). Third, the epileptogenic zone is located based on the result of PAC and analysis of network characteristics (Figure 1C).

### Clinical Data

The long-term SEEG recordings of 17 patients were obtained from Sanbo Brain Hospital of Capital Medical University in Beijing (patient details are shown in Table 1). These patients had been treated with drugs earlier, but the seizures were not effectively controlled, so they had to resort to surgery. For most of the patients, there was no recurrence of seizures more than 2 years after the surgical resection. This study was conducted with the permission of the Ethics Committee of Sanbo Brain Hospital of Capital Medical University with written consent from all patients.

**Table 1:** Clinical features.

**Patient**	**Gender**	**Affected hemisphere**	**Age at onset/surgery**	**SEEG finding**		**Pathology**	**Surgery**
				**Location of seizure onset**	**Type of seizure onset**		
1	M	R	12/38	Me	SpFD	HS	RSAH
2	F	L	12/26	Me	SpFD	HS+GLIOSIS	LSAH
3	F	R	3/26	Me	SpFD	HS	RSAH
4	F	R+L	23/50	Me(R)	LVFA	HS	RSAH
5	M	R	3/14	Me	SpFD	HS	RSAH
6	M	R+L	9/14	Me(L)	LVFA	HS	LSAH+LATL
7	M	L	6/21	Me	LFPS	HS+FCDIb	RATL
8	F	R	17/28	Me	SpFD	HS+FCDIb	RATL
9	M	R+L	16/32	Me(R)	LVFA	HS+FCDIb	RATL
10	F	R+L	4/29	Me	LVFA	HS+FCDIb	RATL
11	M	R	12/22	Me	SpFD	HS+FCDIa	RATL
12	M	L	7/39	Me+Nc		FCDIa	LATL
13	F	R+L	13/23	Me(L)	SpFD	HS+FCDIb	LATL
14	F	R	5/20	Me		HS+FCDIb	RATL
15	F	R+L	3/23	Me	SpFD	HS+FCDIb	LATL+BCFC
16	M	R	15/37	Nc		FCDIb	RATL+BCFC
17	F	R	31/37	Me	SpFD	HS+FCDIb	RATL

*SAH, selective amygdalohippocampectomy; ATL, anterior temporal lobectomy; SpFD, spike or polyspike fast discharges; LFPS, low-frequency high-amplitude periodic spikes; LVFA, low-voltage fast activity; Me, medial; Nc, ectocondyle; FCD, Focal cortical dysplasia; BCFC, bipolar coagulation on functional cortex*.

A series of non-invasive preoperative evaluations are required prior to SEEG monitoring. These evaluations ensure that the implanted electrodes accurately detect the source of the abnormal signal. Electrode implantation was performed by an experienced senior surgeon and an assistant, under general anesthesia with the assistance of the ROSA surgical robot system (24). The number of electrodes per patient is not absolutely fixed, but usually 7–14, and each electrode has 8–16 contacts for local field potential acquisition. The average acquisition period is 1 week.

SEEG recordings containing clinical seizures were selected for each patient in this study, including interictal (5 min), preictal (2 min), and ictal (2 min, the duration of the seizure in some patients is less than 2 min), with the division of each stage given by two electrophysiologists. The SEEG signals acquired in this paper were sampled at 512 Hz or 1,024 Hz with a uniform sampling rate of 512 Hz. The EEGLAB toolbox in Matlab (https://sccn.ucsd.edu/eeglab/index.php) was used to perform the basic preprocessing of the data, such as removing noise and bad channels (Figure 1A).

### Phase–Amplitude Coupling

First, band-pass filtering of the signal x(t) on each channel is performed with the FIR filter (Finite Impulse Response Filter) in EEGLAB, and the frequency bands are divided into delta (0.5–4 Hz), theta (4–8 Hz), alpha (8–13 Hz), beta (13–30 Hz), and gamma (30–80 Hz). Second, low frequency phase sequence *x*_*l*_*pha*_(*t*) and high frequency amplitude sequence *x*_*h*_*mag*_(*t*) on the channel are extracted by Hilbert transform. There is no fixed standard metric for the measurement of PAC. Different metrics are chosen to quantify the coupling strength according to the research contexts (25). In this study, we chose modulation index (MI) to reflect the change of coupling strength, which is overall the best measurement method, not only for data length, sampling rate, and signal-to-noise ratio, but also for other influencing factors in the data.

MI is a method based on a standardized entropy metric to characterize the coupling strength. We refer to the division criteria of Tort et al. (14), as the low-frequency phase with a width of 20° bins (−180°~180°). The average value of the high-frequency amplitude is calculated within each bin (${\bar{x}}_{h\text{\_}mag}$), which is normalized to,


(1) $$\text{P}\left(j\right)=\frac{{\bar{x}}_{h\_mag}(j)}{\sum_{i=1}^{N}{\bar{x}}_{h\_mag}(i)}$$

where *N* is the number of bins, and the range of *j* is [1*,N*]. Shannon entropy is a measure of probability density based on the probability distribution of amplitude values, and here it is used to describe the distribution of amplitudes. Shannon entropy, which gets to maximum when ${\bar{x}}_{h_{mag}}$ in each bin is equal, is calculated as follows,


(2) $$\text{H}\left(\text{P}\right)=-\sum_{j=1}^{N}\text{P}\left(j\right)\log\text{P}(j)$$

**Figure 2:**
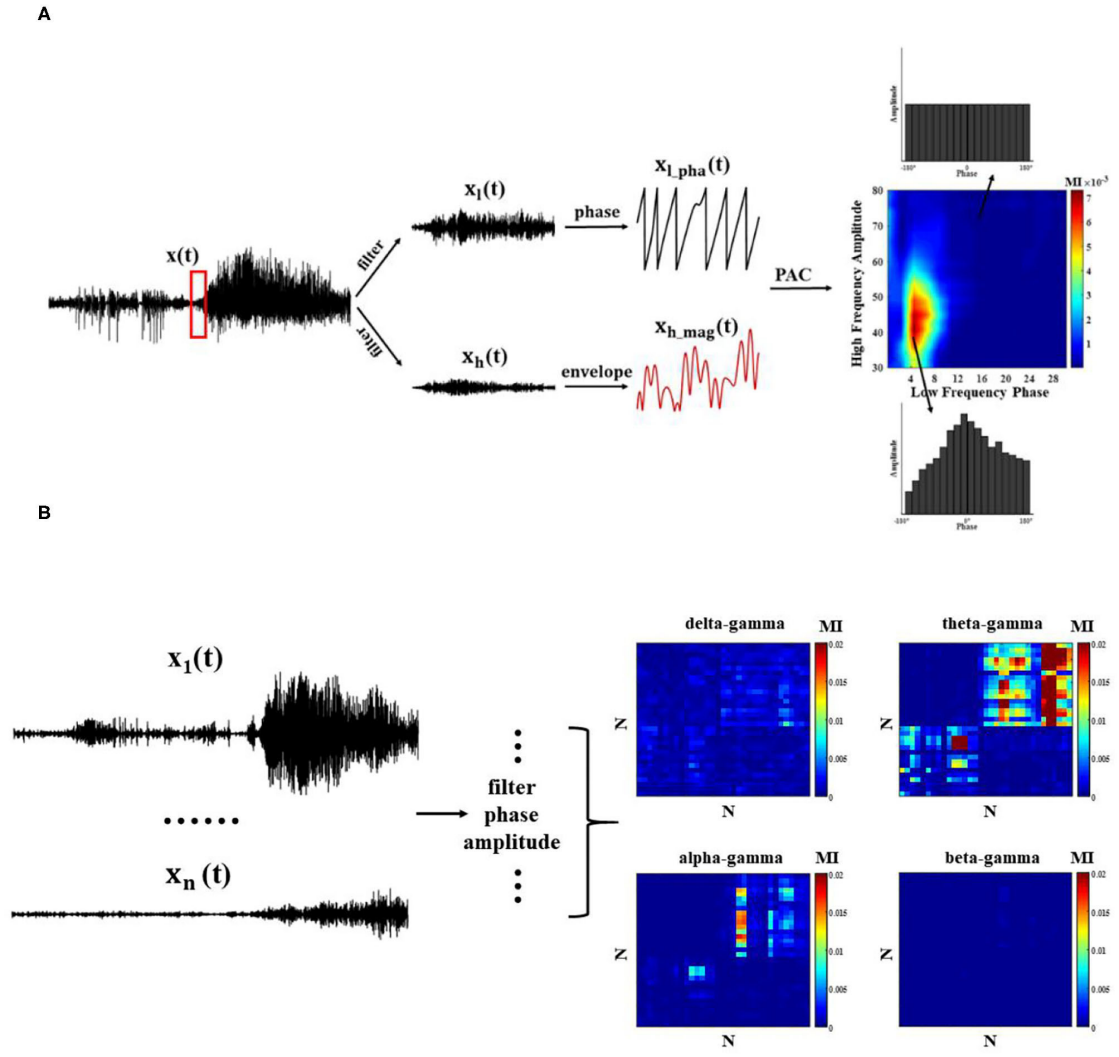
Phase–amplitude coupling (PAC) quantization method. **(A)** Single-channel PAC: each channel undergoes bandpass filtering and extracts the corresponding phase and amplitude envelope time series, and MI index measures PAC; **(B)** Cross-channel PAC.


When the phase amplitude coupling phenomenon exists, P(*j*) deviates from the uniform distribution (Figure 2A). We use the Kullback–Leibler metric to measure the difference between the distribution P and the uniform distribution,

(3) KL (U, P) = log N−H(P),

where U is uniform distribution, and log*N* is a maximum entropy value. MI is calculated as,


(4) $$\text{MI}=\frac{\text{KL}(\text{U},\text{P})}{\log N}$$

Most previous studies have discussed the characteristics of intra-phase amplitude coupling within a single channel, which helps determine the period of attack or the location of the EZ (19, 20, 26). In this paper, we use the features of intra- and cross-channel PAC within a single channel to determine the location of the EZ. For single-channel, the low-frequency phase and high-frequency amplitude envelope information within each channel are extracted using the method mentioned above, and the MI index is used to calculate the PAC values of different band pairs throughout the episode. As the channels record the electrical activity in different brain regions, the MI values are analyzed after normalization using z-scores. Z-scores are often used to filter the data for abnormal values, which are considered abnormal when they are >3 typically (27, 28).

To observe the modulation effect between different regions at the network level, it is also necessary to calculate the PAC across channels (Figure 2B). Next, we compute the average cross-channel coupling matrix (*N* x *N*) under each window to understand the diversity of coupling band pairs over time.

### Network Analysis

In recent years, the study of static and dynamic brain networks has developed rapidly. Advances in graph theory and network neuroscience have provided opportunities to understand the details of this complex phenomenon and its modeling. The graph theory approach establishes a mathematical framework to simulate paired communication between network nodes. In addition, it also can be applied to functional and structural connections in neuroscience. Graph-based network analysis reveals meaningful information about the topology of human brain networks, such as small worlds, modular organizations, and highly connected or centralized centers.

In this study, we use a sliding window approach to build dynamic functional connectivity networks, and the window size corresponds to 10 s. The elements in the connectivity matrix represent the phase–amplitude coupling strength (MI) between two channels. At a certain threshold T, the MI graph obtained by cross-channel PAC is transformed into an adjacency matrix. When the element (MI) in the matrix is greater than the threshold T, we consider that there is modulation between the channels, otherwise the connection is removed. At different thresholds, the network can exhibit very different properties. If the threshold value is not selected properly, there will be a big difference in the number of connected edges under different networks, which will affect the subsequent study of the network characteristics. Therefore, k-degree is proposed to help determine the network connectivity, which refers to the average number of connected edges that exist in the network (29, 30). Performing network analysis at a determined k-degree can help avoid inaccurate features caused by large connected edge differences. The k-degree is a function of the threshold T, and there will be a corresponding k-degree under each threshold. To ensure that this number can be used to identify the network features and variations of the network over time, several sets of k-degree are tested. The k-degree is usually chosen between 3 and 7. As there are too many network nodes extracted in this study, the k-degree is controlled between 1 and 2 in this study to better observe the changes in network connectivity edges.

After the network connection matrix is determined, indices of graph theory, such as node degree, characteristic path length, and cluster coefficient, are used to analyze the network characteristics. We use the clustering coefficient (C), characteristic path length (L), and betweenness centrality under different time to analyze the characteristics of the brain function network (Figure 1C) (31, 32). It is important to note that usually the average path length is calculated in a connected network, but the actual network is often disconnected. If there is no connected path between two nodes, which will result in an infinite distance between nodes, an infinite average path length for the whole network appears. To avoid this divergence problem, we define the average path length of the network as the average of the distances between pairs of nodes with connected paths.

## Result

### Single-Channel Phase Amplitude Coupling

**Figure 3:**
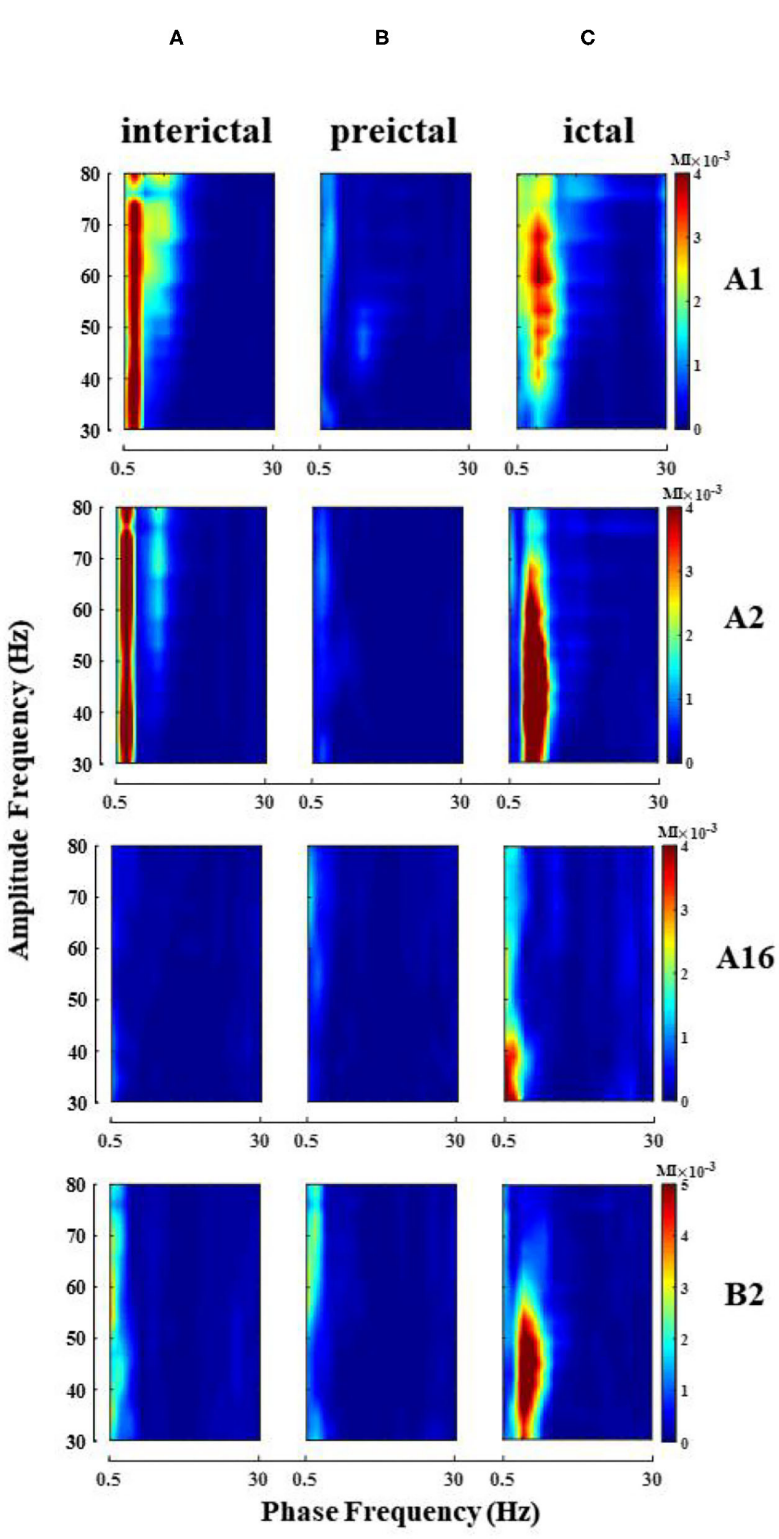
Single-channel PAC changes during seizure (patient 2). Electrode A1–16 (left amygdala-middle temporal gyrus, some channel results are shown), electrode B2 (left hippocampus). **(A)** Interictal; **(B)** Preictal; **(C)** Ictal, the phase of theta and amplitude of gamma coupling increase (Note that the magnitude of coupling varies between channels).

Phase–amplitude coupling was calculated for all electrode channels in different seizure periods of each patient, and theta–gamma PAC was significantly enhanced in some electrode channels during the ictal (Figure 3 shows the change of PAC in some channels of patient 2), but not always in the strong coupling state. At the same time, due to the different locations of the electrodes implanted in various brain regions, we observed some differences in the intensity and scale of PAC in different channels in the single-channel PAC results. Take note that the enhanced coupling mentioned above is for the whole seizure process in the single channel.

**Figure 4:**
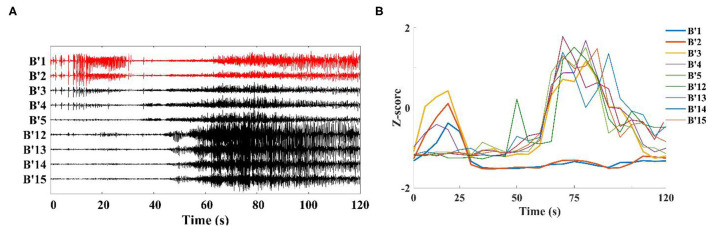
Channels on the middle temporal gyrus-hippocampal head electrode (patient 1). **(A)** SEEG recording at the ictal; **(B)** Modulation index of each channel is converted to Z-score during the ictal, first to show coupling changes were B'1**–**B'3 in this electrode.

When analyzing the SEEG of the patient during the ictal period, we found that the time points of abnormal discharge were different for different channels. As shown in Figure 4A, abnormal discharges appeared in channels B'1**–**B'3 (Right hippocampus) of patient 1 at the beginning of the seizure period, while similar abnormal discharges appeared in the remaining contact channels on this electrode only 40 s after the seizure. For this phenomenon, we analyzed the onset period using a sliding window analysis with a window size of 10 s. The results showed theta–gamma PAC increased in channels B'1–B'3 at the beginning of the seizure, but then dropped to the interictal intensity level during middle- and late-ictal. The similar phenomenon occurred on the remaining channels, but at slightly different time (Figure 4B). We compared the channels with the above characteristics for each patient with the corresponding clinically diagnosed epileptogenic zone and found significant overlap between the two types of channels. The range of brain regions involved in the abnormal channels identified by a single channel in this paper will be relatively wide.

### Cross-Channel Phase Amplitude Coupling

**Figure 5:**
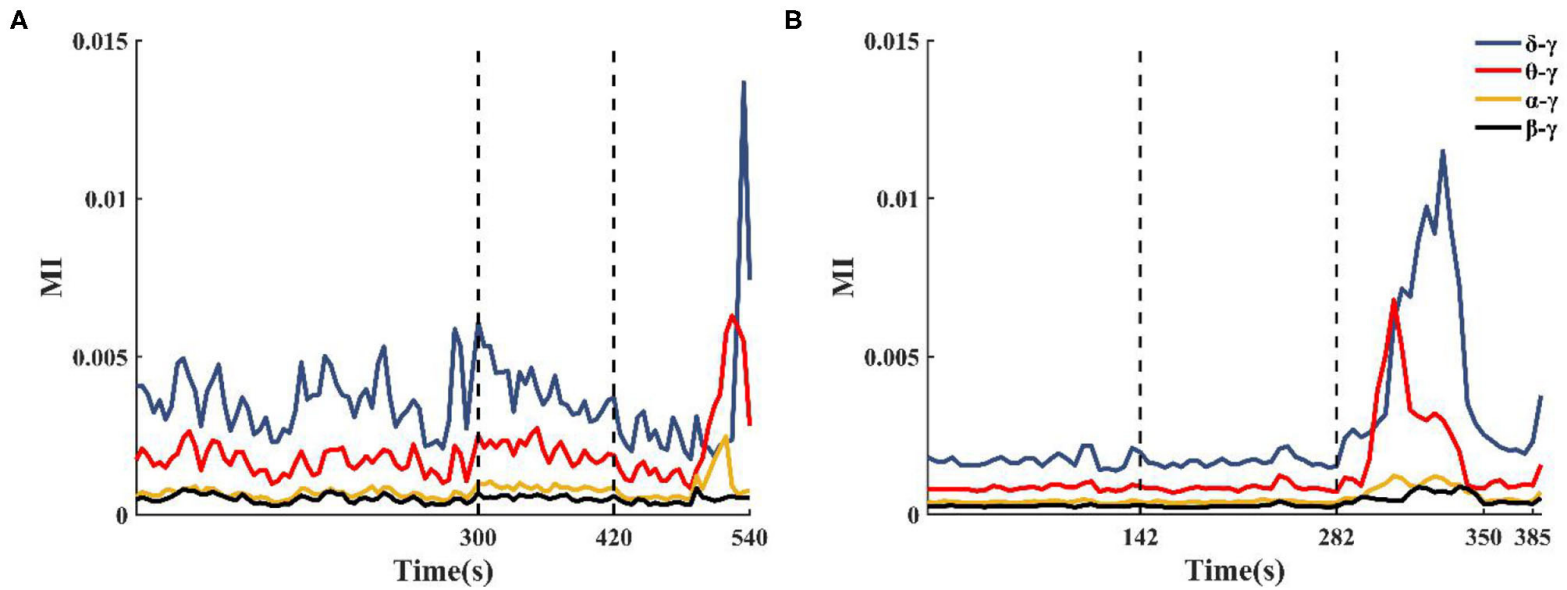
Changes in cross-channel PAC frequency band characteristics during seizures. **(A)** Patient 2 seizure is not over at 540 s, **(B)** Patient 13 seizure is over at 350 s.

The enhanced theta–gamma PAC allowed us to screen out some potential channels of epileptogenic zone in single-channel coupling analysis. However, the range involved in these channels is still too large. To obtain changes in the coupling characteristics of the epileptogenic channels during the seizure, based on the results of the clinical analysis of the patient's SEEG and the preliminary results of the single-channel analysis, some channels from the implanted electrode channels were selected for cross-channel PAC (the number of channels N was controlled at 30~50). The coupling strengths of low-frequency band (delta, theta, alpha, beta) phase and high-frequency band amplitude (gamma) between channels were calculated at different onset periods. The results showed that the alpha–gamma and beta–gamma PAC did not fluctuate much with seizures, and the delta–gamma and theta–gamma coupling intensities had significant peaks during the seizure period (Figure 5). Combined with the calculation results of the remaining patients, we concluded that the phase amplitude coupling of theta-gamma is significantly enhanced during the seizure period. We also found that the delta–gamma PAC in patients, whose seizures ended within 2 min of the ictal period, had a sudden increase in the middle- and late-ictal. Delta–gamma was more significantly changed than theta–gamma. The duration of the seizure period for each patient collected in this paper was 2 min, but individual patients had greater seizure duration. Therefore, we do not discuss much about the characteristics of delta–gamma during the mid-late ictal (Figure 5A).

### Localizations of Epileptogenic Zone

In single-channel PAC analysis, we found that the channels with the phenomenon of increased theta–gamma PAC during seizures overlapped with the abnormal channels localized in the clinical diagnosis of SEEG. Although the channel range determined by our single-channel coupling analysis has been somewhat reduced compared to the SEEG implanted electrode channel range (by about 50%), to more precisely locate and understand the changes in the epileptic network during seizures, cross-channel PAC between theta and gamma bands is calculated. We use clustering coefficients, path lengths, and betweenness centrality to extract network features.

**Figure 6:**
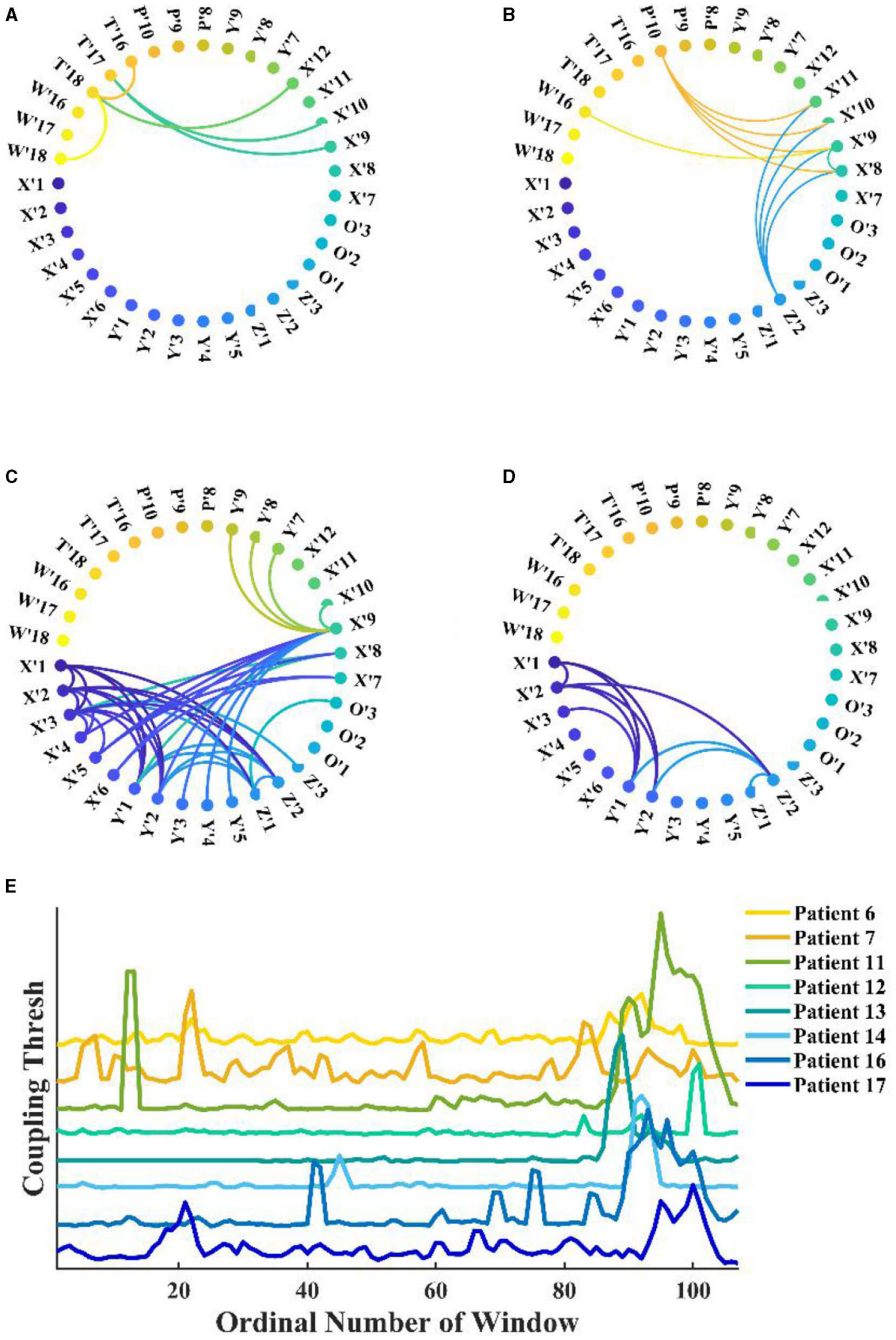
Functional network from patient 11 during seizure. **(A–D)** Interictal, early-ictal, middle-ictal, and late-ictal. Channels X'1–O'3 are the medial cerebral channels and channels X'7–W'18 are lateral cerebral channels; **(E)** Seizure network coupling thresholds vary at fixed k-degrees, seizure onset times are marked by arrows in the figure (specific seizure times are not marked), and these patients had complete seizure periods.

Under fixed threshold conditions, significant changes were found in the network connectivity between seizure periods. Figures 6A–D shows the network for patient 11, where we controlled the threshold at 0.04, with the most connected edges occurring in the mid-ictal period and concentrated between the patient's medial channels. During the interictal period, there were only five connections larger than the threshold. By fixing the k-degree, one can observe the variations with the seizure network threshold. In Figure 6E, we found that the threshold for the network increases and then decreases with the seizure in patients with complete seizure onset. It reflects the change in the strength of theta**–**gamma phase amplitude coupling between channels.

**Figure 7:**
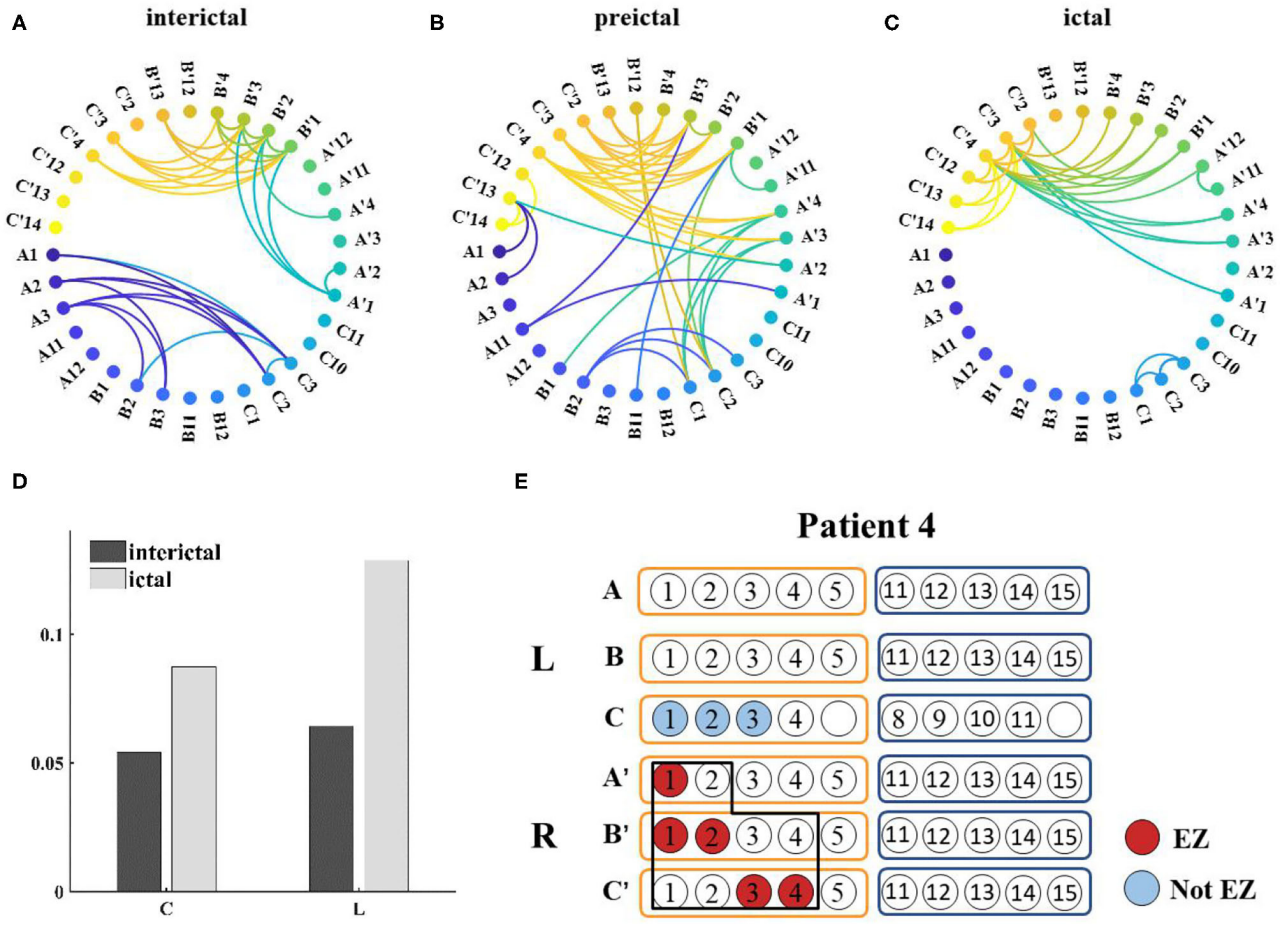
**(A–C)** Functional network from patient 4 during seizure (k-degree = 1.3), threshold values are 0.0012, 0.007, and 0.0196, respectively; **(D)** Distribution of network characteristics under different attack periods, the clustering coefficient (C) and the mean path length (L) increased during the ictal; **(E)** Prediction of epileptogenic zone (EZ) and surgical resection location. The red marker represents the main EZ localized and the blue marker represents the area affected by PAC, and the black box represents the surgically resected area.

**Figure 8:**
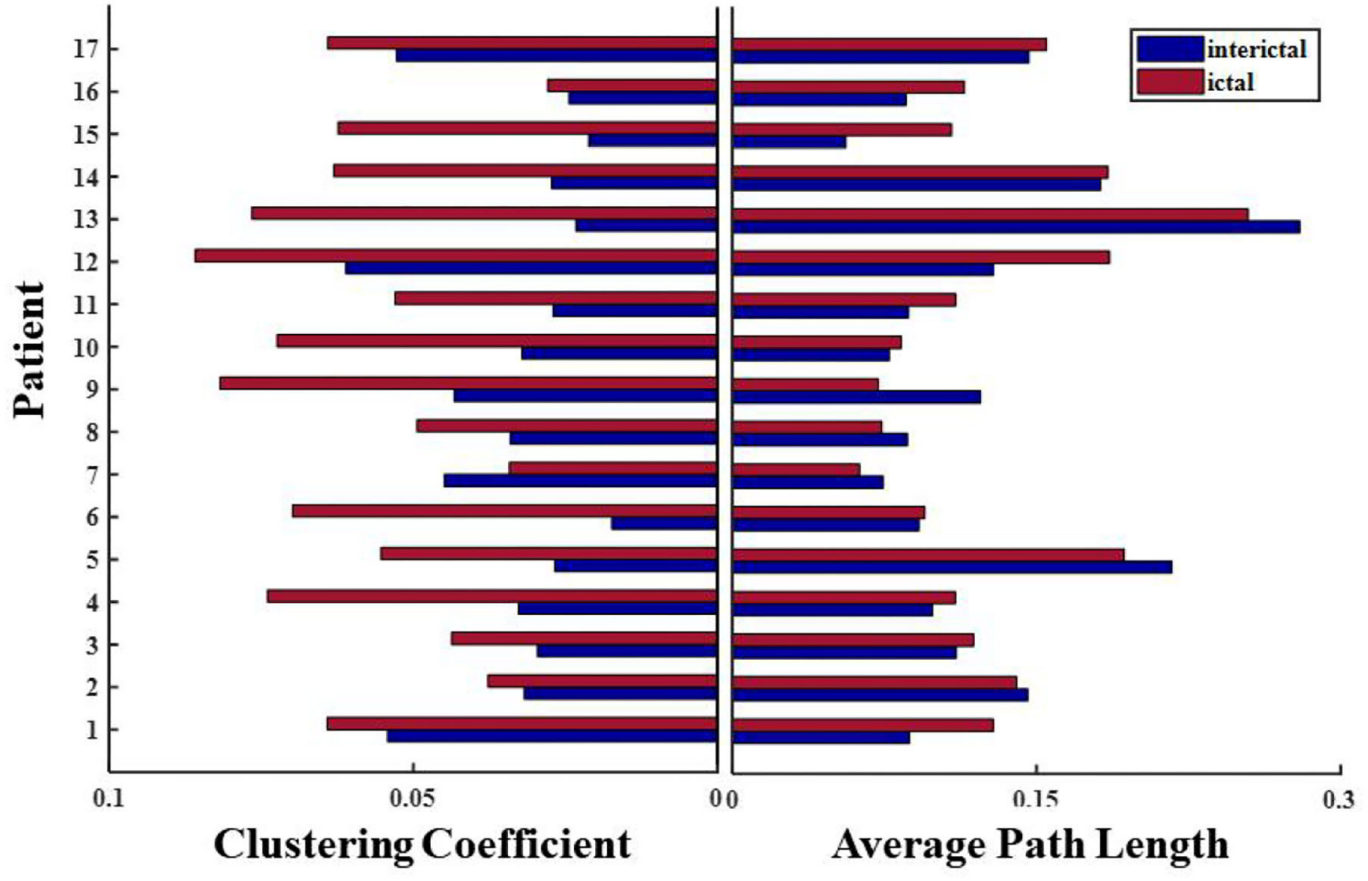
Changes in network characteristics in patients with different seizure periods.

Network connections extracted based on PAC with time are depicted for patient 4 in Figure 7. Based on the SEEG data and clinical diagnosis, we understand that patient 4 had electrodes implanted on both the left and right side of the brain, and abnormal discharges were observed bilaterally, with the right side standing out. In the extracted local network connectivity structures under different seizure periods, it was observed that at a fixed k-degree, no coupling relationship existed between the left and right lateral channels during the interictal, which was mainly present on the respective side of the brain, and the coupling strength was weak. During the preictal period, connections between the left and right lateral channels emerged and the connectivity was enhanced. However, we observed that the strong coupling between the right channels remained, and the coupling between the left channels decreased. During the ictal period, the coupling intensity between the channels was highest on the side of the brain where the EZ was located (Figure 7C). In Figure 7D, both clustering coefficients (C) and mean path length (L) increase at the ictal, and the network is more regular during the ictal period than the random network during the interictal period (Figure 8 shows the results for clustering coefficients and mean path length for all patients).

Based on this analysis, results for the remaining patients can be found in Table 2. The calculated onset channels were identical to the channel locations marked by the SEEG report, and the accuracy rate of the epileptogenic zone (EZ) was ~82.4% (the detectable rate of EZ was 89.7%). In some of these individual cases, the calculated epileptogenic zone was a little different from the surgical plan. It could be categorized into three main categories: (1) Patient 9 and 13 were seizure-free postoperatively but experienced involuntary movements. The calculations showed abnormalities bilaterally, with one side being more significant. The surgical plans were taken to remove the epileptogenic zone on one side, corresponding to the location of the area on the side where the PAC was significant in the calculation; (2) Patients 10 and 12 underwent resection of the lateral and medial temporal lobe structures and had good postoperative results. However, our calculations showed only the medial channel abnormalities and did not identify the lateral channels. There is some discrepancy with the surgical protocol; (3) Patient 6 underwent two surgeries for selective amygdalohippocampectomy (SAH) and anterior temporal lobectomy (ATL). The left medial and lateral structures were resected successively. However, the patient had another seizure 8 months after the second surgery. The calculations showed abnormalities in the left hippocampus, amygdala, and right hippocampus of patient 6, and no abnormal left lateral channels were identified. There is some discrepancy with the surgical protocol (Patients with ^*^ in Table 2 are those for whom the SAH procedure was recommended based on the calculated results, which differ from the actual surgical protocol ATL).

**Table 2:** Calculated results compared with clinical conclusions.

**Patient**	**Focal analysis results**		**SEEG report**	**Resection (clinic)**	**Outcome**
	**EOZ**	**EZ**			
1	B'1–2	HIP.R	B'1–3	ME-R	I
2	A1–2, B1–2	AMYG.L, HIP.L, PHG.L, Foci	A1–3	ME-L+Foci	I
3	C'1, B'11–13	HIP.R, AMYG.R	B'2–4	ME-R	I
4	B'1–3, C'3	HIP.R, AMYG.R	A'1–2	ME-R	I
5	B'1	HIP.R, AMYG.R	B'1–3	ME-R	I
6*	A1–2, B2	AMYG.L, HIP.L, (HIP.R)	A1–2	ME+Nc-L	II
7	C1–2, B'3	HIP.R, MTG.R, TPOmid.R	A'2–3	ME+Nc-R	I
8	A'1, B'2	HIP.R, AMYG.R, MTG.R	A'1	ME+Nc-R	I
9	A11–12, B'2	HIP.R, MTG.R, (HIP.L)	B'2–3	ME+Nc-R	I
10*	B'1–3	HIP.R, (AMYG.L)	B'1–2	ME+Nc-R	II
11	X'4–6	TPOmid.R, HIP.R	X'11–12	ME+Nc-R	I
12*	G1–2	HIP.L, AMYG.L	G2	ME+Nc-L	II
13	H14	AMYG.L, INSULA, TPOmid.L, HIP.L; (HIP.R)	H1–3	ME+Nc-L	I
14	B'1–4, A'1	HIP.R, AMYG.R	B'1–3, A'1–2	ME+Nc-R	I
15	A9–12	TL.L, HIP.L, INSULA	A8–9,11–12	ME+Nc-L+BCFC(HIP)	I
16	A'1–3	HIP.R, AMYG.R, INSULA, (STG.R)	D'4–5	ME+Nc-R+BCFC(INSULA)	I
17	A'1, B'1	HIP.R, AMYG.R, SMG.R, MTG.R	A'1–2	ME+Nc-R	I

*EOZ, epileptic onset zones; HIP, hippocampus; AMYG, amygdala; PHG, parahippocampal gyrus; MTG, middle temporal gyrus; TPOmid, temporal pole middle temporal gyrus; TL, temporal lobe; STG, superior temporal gyrus; SMG, supramarginal gyrus; ME, medial; Nc, ectocondyle; BCFC, bipolar coagulation on functional cortex; I, Consistent with surgical plan; II, Inconsistent with surgical plan*.

## Discussion and Conclusion

In this study, cross-frequency PAC is used to analyze the coupling characteristics over different brain regions during seizures in temporal lobe epilepsy patients to help localize the epileptogenic zone. The electrode channels with increased PAC at theta–gamma and key channels in the dynamic functional network overlap significantly with the clinically defined EZ. Furthermore, based on the dynamic functional network extracted by cross-frequency PAC, we found that the network became more regular during the ictal, and the seizure features spread from local to global and finally focus on the EZ. In conclusion, it is found in this study that identifying channels with theta–gamma phase amplitude coupling increasing with seizures and combining single-channel results with multi-channel results can better localize the epileptogenic region.

While the initial focus of EEG research on rhythms was on the physiological relationships that explain the normal activity, in recent years it has been used mainly in clinical diagnosis. There is growing evidence that neural rhythm oscillations have unique coupling properties and cross-band coupling phenomena occur in different frequency bands, brain regions, and under various task conditions. Schack et al. (33) found that the phase of the theta frequency modulates the amplitude of the gamma through bispectral analysis of experimental data when the human brain is engaged in perceptual and memory tasks, i.e., there is PAC. Canolty et al. (34) found that in auditory and memory experiments, PAC was present in most of the EEG signals measured by subdural electrodes, and PAC was present in all regions of the brain. Weiss et al. (26) showed that the PAC between high-gamma and low-frequency increases in the later-ictal period and, in particular, the topographical features of the phenomenon predicted resection success. Such studies have discussed the PAC changes during the seizure in specific regions of the channel, whereas the study in our paper is an analysis of the PAC characteristics of all the channels from interictal to ictal. In some channels, coupling on theta–gamma increased during the ictal period, and the number of channels identified by this feature accounts for about 30–45% of the channels in the data set. The placement of the SEEG electrodes in each patient is based on the results of various preoperative examinations. It detects the area of possible EZ. We further narrowed down the possible EZ by single-channel analysis. However, the channels of some patients correspond to a slightly wider range of brain regions than the surgically removed area. Only relying on a single channel cannot accurately locate the main epileptogenic area.

Seizures originate from “epileptic networks,” in which the combination of multiple neurons can trigger seizures from multiple locations and evolve into stereoscopic, macroscopic seizures (35). When an abnormality occurs in one node of the brain network, the area with which a connection exists is affected, and eventually the subnetwork or even the whole network is disrupted (31). Coito et al. (36) investigated the changes in the interictal network in patients with unilateral temporal lobe epilepsy. They found right temporal lobe epilepsy (RTLE) has strong connections from the ipsilateral to the contralateral medial side, which affect the normal activity of the contralateral side. It was also indicated that an enhanced model of dynamic connectivity patterns could better aid in the diagnosis of epilepsy. Wang et al. (23) found in their studies on memory encoding that inter-regional PAC may be a mechanism, reflecting the coordinated relationship between brain regions. To better locate the EZ, we analyzed cross-channel coupling based on single-channel PAC. The changes in the characteristics of the network during seizures were observed in the functional network obtained under continuous sliding windows. We then used the betweenness centrality to calculate the centrality of each node in the network.

The reason why the study chose to analyze on a single-channel basis is that, first, it reduces the computing effort. The data analyzed in this study is SEEG recording. Due to patient specificity, there are variations in the regions and on the number of electrodes implanted. The number of channels with the least number of regions detected is also >50. Extensive time and effort are consumed to calculate the coupling between all channels. Second, precise localization is performed. Some studies showed that EZ has a strong coupling status (19, 21), and single-channel analysis can help to further narrow down the EZ. Patient 4 is a right temporal lobe epilepsy patient whose SEEG recordings showed bilateral abnormal discharges. Single-channel PAC results showed the presence of theta–gamma PAC enhancement in some of the channels bilaterally. This result does not indicate where the EZ is primarily located. Moreover, in combination with the multi-channel results, it was observed that the seizure network showed strong connections, mainly in the right hippocampal–amygdala region. The nodal centrality analysis also showed that the patient's main focal area was on the right medial side, and right selective amygdalohippocampectomy (RSAH) was recommended. This was consistent with the patient's clinical record results and seizure-free condition after surgery. In case of patient 2, abnormalities are mainly shown in the medial hippocampus and parahippocampal gyrus during the single-channel analysis, while new abnormal areas, focal areas (low-grade tumor areas) are found in the multi-channel analysis. Selective resection of the hippocampus and the focal portion of the EZ on the left side can be chosen, which is consistent with the comparison of the postoperative results.

The combination of single-channel and multi-channel PAC can better help identify EZ. It can more intuitively get the changes in the network characteristics of brain functional networks during seizures, such as the transient shift to regular networks during ictal. However, there are also some shortcomings in this study. We mainly focus on the theta–gamma, which has a significant relative change during the seizure. However, other frequency band pairs are not analyzed, such as the sudden increase of delta–gamma PAC during the cross-channel analysis. Later, other coupled frequency band pairs can be discussed to determine whether they can help better locate or predict the attack state.

The recent study focuses on the identification of seizure state and EZ in epilepsy based on the analysis of clinical data. The integration of frequency coupling relationships between different brain regions in the form of functional networks is of great help in guiding the study of complex action relationships on neuronal modeling and neuronal populations. Moreover, the study of mechanisms that modulate different seizure states through the strength of PAC is crucial. It helps to use the model for future studies on spatial scales, from oscillatory activities at the neuronal level in seizure states to functional coupling of brain rhythms at the systematic level.

## Data Availability Statement

The original contributions presented in the study are included in the article/supplementary material, further inquiries can be directed to the corresponding author/s.

## Ethics Statement

This study was carried out in accordance with the recommendations of the Ethics Committee of the Sanbo Brain Hospital of Capital Medical University. All subjects gave written informed consent in accordance with the Declaration of Helsinki. The protocol was approved by the Ethics Committee of the Sanbo Hospital of Capital Medical University. Written informed consent to participate in this study was provided by the participants' legal guardian/next of kin. Written informed consent was obtained from the individual(s) for the publication of any potentially identifiable images or data included in this article.

## Author Contributions

XL, FH, RF, QW, and GL collected, processed, analyzed epilepsy data sets to locate epileptogenic zone, and wrote the paper. All authors contributed to the article and approved the submitted version.

## Funding

This research was supported by the National Science Foundation of China (Grant Nos. 11932003 and 11972115).

## Conflict of Interest

The authors declare that the research was conducted in the absence of any commercial or financial relationships that could be construed as a potential conflict of interest.

## Publisher's Note

All claims expressed in this article are solely those of the authors and do not necessarily represent those of their affiliated organizations, or those of the publisher, the editors and the reviewers. Any product that may be evaluated in this article, or claim that may be made by its manufacturer, is not guaranteed or endorsed by the publisher.
